# Bushen Wenyang Huayu Decoction Targets TLR4/NF-*κ*B Mediated Autophagy to Treat Endometriosis Effectively

**DOI:** 10.1155/2022/4263417

**Published:** 2022-11-18

**Authors:** Ying Li, Xin Meng, Xinping Fu, Mingli An, Huimin Liu, Yiming Ma, Qingxue Li, Guorong Hao, Yucong Ma, Yu Zhang, Jian Yang, Jingwei Chen

**Affiliations:** ^1^Hebei Key Laboratory of Integrative Medicine on Liver-Kidney Patterns, Institute of Integrative Medicine, Hebei University of Chinese Medicine, Shijiazhuang 050200, China; ^2^Department of Gynecology, The Fourth Hospital of Shijiazhuang, Shijiazhuang 050011, China; ^3^Department of Rehabilitation, Youfu Hospital of Hebei Province, Shijiazhuang 050051, China

## Abstract

Endometriosis has been found to be closely related to autophagy. This study aimed to elucidate the possible mechanism of Bushen Wenyang Huayu Decoction (BWHD) in treating endometriosis (EMs) by targeting TLR4/NF-*κ*B-mediated autophagy. Autologous grafting was used to generate the EMs model in rats. Once the model was developed, BWHD high-dose and low-dose groups received intragastric administration of BWHD, and the gestrinone group served as a positive control. Immunofluorescence labeling and Western blotting were used for the protein expression of toll-like receptor 4 (TLR4), nuclear transcription factor-*κ*B (NF-*κ*B), Beclin-1, and selective autophagy connector protein P62 (P62). Quantitative real-time polymerase chain reaction (qRT-PCR) was used to analyze mRNA levels of TLR4, NF-*κ*B, Beclin-1, and P62. We found that BWHD significantly reduced the size of ectopic lesions in rats with EMs, regulated reproductive hormone levels, and alleviated the cell autophagy level. It suggested that BWHD could be an effective treatment of EMs by targeting TLR4/NF-*κ*B signaling pathway.

## 1. Introduction

Endometriosis (EMs) is a chronic condition that causes discomfort and disability and affects about 15% of women of childbearing age; it is also believed to afflict 30%–50% of infertile women [1]. Aberrant proliferation, infiltration, and recurrent bleeding of stromal cells and endometrial epithelial cells outside of the uterine cavity lead to the formation of nodules and masses, which can result in a wide range of clinical symptoms, including chronic pelvic discomfort, dysmenorrhea, menorrhagia, and infertility [2]. EMs is a frequent gynecological condition with a high recurrence rate [3], which has a detrimental effect on the quality of life for those affected by it. Accordingly, more research into EMs is required to address this pressing problem better [4]. Despite extensive research, the etiology and pathogenesis of EMs remain unclear; however, several studies have shown that the occurrence of EMs may be related to autophagy [5–7].

Autophagy is a highly conserved cellular process involved in decomposition, metabolism, and recycling, which plays a pivotal role in maintaining cellular homeostasis as well as the normal function of organelles [8, 9], and autophagy has been linked to several human diseases, such as Parkinson's disease, metabolic diseases, EMs, and cancers [10–14].

Autophagy is believed to act as a housekeeping regulator to keep the intracellular environment stable by degrading and recycling damaged or unneeded parts [15]. Hypoxia, endoplasmic reticulum stress, starvation, cytotoxicity, and infection are all regulators that influence the process of autophagy [16]. However, despite abundant literature about autophagy, its role in EMs is still controversial. Several reports have suggested that EMs has increased autophagy [17–19]; however other researchers have come to the opposite result [7, 20]. It is important to find novel strategies to cure EMs by modulating autophagy.

TLR4/NF-*κ*B constitutes the classical inflammatory signaling pathway, can play crucial roles in mediating immune and inflammatory responses, and participate in the pathological process of EMs [21, 22]; furthermore, autophagy was thought to be regulated via TLR4/NF-κB signaling pathway [23, 24]. Recently, TLR4/NF-*κ*B has been implicated in EMs due to the critical regulatory roles of inflammation, indicating that targeting TLR4/NF-*κ*B might be a strategy for EMs therapy. However, it has not been reported to detect the induction of autophagy by TLR4/NF-*κ*B in EMs. Therefore, this study aimed to investigate the role of TLR4/NF-*κ*B in EMs and its potential mechanism.

The importance of TCM's efficacy in the management of EMs has emerged in recent years [25, 26]. Previous research by our team demonstrated that BWHD has great clinical effectiveness in treating EMs and can play a crucial role in supporting the atrophy of ectopic lesions by influencing many channels and targets [27]. In this study, the potential mechanisms of BWHD on TLR4/NF-*κ*B signaling pathway and the levels of autophagy-related factors Beclin-1 and P62 were observed to clarify the mode of action of BWHD for EMs treatment, thus providing a useful theoretical basis for deep application in this disease.

## 2. Materials and Methods

To perform this study, a total of 100 SPF female SD rats aged between 8 and 10 weeks and weighing 220 ± 20 g were purchased from the Beijing Weitong Lihua Experimental Animal Technology Co., Ltd. (SCXK (Beijing) 2016-0011). The experiment was carried out in the Scientific Research Center of Hebei University of Chinese Medicine (SYXK (Hebei) 2017-005). Good ventilation and adaptive feeding were used during the one-week experiment, including the regular pellet meal and drinking water at room temperature (23 ± 2°C). All the different procedures were performed according to the ethical standards of the Ethics Committee of Hebei University of Chinese Medicine (no. DWLL2020031).

### 2.1. Experimental Drugs

BWHD consists of Zhifuzi (Radix Aconiti Lateralis Praeparata) 6 g, Rougui (Cortex Cinnamomi) 10 g, Xiaohuixiang (Fructus Foeniculi) 10 g, Yanhusuo (Rhizoma Corydalis) 15 g, wuzhuyu (Euodia sp.) 9 g, SangJiSheng (Ramulus Taxilli) 30g, GouJi (Woodwardiajaponica) 30 g, and so on. These herbals were purchased from Shijiazhuang Lerentang Pharmaceutical Co., Ltd. and decocted in water. BWHD high-dose and low-dose containing 1.85 g/ml and 4.625 g/ml of the drugs were prepared and stored at 4°C.

Estradiol valerate tablets were purchased from Bayer Medical Care Co., Ltd., Guangzhou Branch, batch no. 469A, Gestrinone capsule from Beijing Zizhu Pharmaceutical Co., Ltd., batch number: 53190501, while isoflurane was purchased from the Beijing Keyue Huacheng Technology Co., Ltd., batch number: 201805.

### 2.2. Reagents

Reagents used were CA125 IRMA kit (Tianjin Xiehe Medical Science & Technology Co., Ltd., cat. no. RC70417); E_2_, P, FSH, and LH ELISA kits (Cayman Chemical, Ann Arbor, MI, USA, cat. no. 501890, 582601, 500710, and 500720); TLR4 antibody (Santa Cruz Biotechnology, Santa Cruz, CA, USA, cat. no. Sc-293072); NF-*κ*B/P65 antibody (Abcam, Cambridge, UK, cat. no. AB16502); Beclin-1 antibody (ProteinTech, Wuhan, China, cat. no. 11306-1-AP); SQSTM1/P62 antibody (Bioss, Beijing, China, cat. no. Bs-55207r); GAPDH (Servicebio, Wuhan, China, cat. no. P04406); and HRP-labeled Goat Anti-Rabbit IgG (Servicebio, Wuhan, China, cat. no. GB23303).

### 2.3. Instruments

Small animal anesthesia machine (U.S. MIDMARK Company, MATRX, VMR); pathology graphic analysis system (Japan, DP72CCD); gel imager (Image Quant, LAS 4010); real-time fluorescence quantitative PCR instrument (Bio-RAD, CFX96), and laser scanning confocal microscope (Leica Microsystems Shanghai Trading Co., Ltd., Leica SP8) were used for the various experiments.

### 2.4. Grouping and Treatment

Following a week of adapted feeding, 100 SPF female SD rats were randomly assigned to 5 groups with 20 rats each: Group 1 = sham group, Group 2 = model group, Group 3 = BWHD high-dose group, Group 4 = BWHD low-dose group, and Group 5 = gestrinone group. Except for the sham group, the EMs model was established by autologous transplantation as described in the literature [28]. All rats had their estrus cycles synchronized three days before the modeling by injecting them with 1 mg/kg of estradiol valerate.

After administering isoflurane inhalation anesthesia and using iodine for standard disinfection, a 2.5 cm ventral midline incision was done to expose the uterus. After ligating the left horn of the uterus, the organ was preserved in a solution of normal saline. The uterine horn was slit longitudinally and then sutured diagonally to the internal right abdominal wall using 4–0 absorbable thread. In the sham group, the left uterine horn was removed, and the abdomen was stitched shut. To prevent an infection from developing, gentamicin (0.1 ml) was administered intraperitoneally three days following the operation. Intragastric administration (1 ml/100 g) was started on the first day after the surgery. The therapeutically equivalent dose was determined to serve as a standard dose (a factor of 6.3 of the adult unit body weight). The high-dose group received 46.25 g/kg of BWHD (2.5 times the clinical equivalent dosage), while the low-dose group received 18.5 g/kg of BWHD (clinical equivalent dosage), once daily. The gestrinone group received 0.25 mg/kg of gestrinone twice a week, and an equal volume of pure water intragastric administration at other times. The model and sham groups were given an equal volume of pure water for three weeks.

### 2.5. Specimen Collection

The size and shape of the ectopic foci in the rats were observed after the administration of anesthesia. The uterus and ectopic foci were surgically removed promptly. To preserve the tissue, some of it was placed in a 4% paraformaldehyde solution, some in a 2.5% glutaraldehyde fixative solution, and the remainder were instantly frozen in liquid nitrogen and stored at a temperature of -80°C.

### 2.6. Measuring of Serum CA125, E_2_, P, FSH, and LH

The serum CA125, E_2_, P, FSH, and LH concentrations were detected by IRMA and ELISA, according to the instructions included in the purchased kits.

### 2.7. Immunofluorescence Staining

Tissue chips were dewaxed with xylene, dehydrated with gradient alcohol, and then subjected to antigen repair and PBS washing. The agents A and B naturally quench fluorescence were added. After washing with PBS, 0.5% Triton-PBS and 10% goat serum were added in jars and sealed correctly. After incubation, the wash was given with PBS and sealed in the pot along with an antifluorescence quenching agent. After 0.5% Triton PBS and PBS washing, 4',6-diamidino-2-phenylindole (DAPI) was incubated for 15 min. Leica SP8 laser scanning confocal microscope was used to observe the various images (Leica, Wetzlar, Germany).

### 2.8. Transmission Electron Microscopy

Tissues were fixed right away in 4% glutaraldehyde at 4°C for 2 hours, then fixed again in 1% osmium acid for another 2 hours. After washing the fixed tissue with 0.1 M PBS, the tissue was embedded in a 1 : 1 acetone/812 mixture for 2–4 hours, a 1 : 2 acetone/812 combination overnight, and a pure 812 embedding agent for 5–8 hours before being dehydrated in ethanol and acetone gradients after dehydration resin block sections were cut with a Leica UC7 ultramicrotome of 60–80 nm (Leica, Wetzlar, Germany). After treating the samples with 2% uranyl acetate-saturated aqueous solution and then treating them with citrate solution for 15 minutes, the ultrastructural alterations were analyzed by looking at the samples with a Hitachi HT7700 transmission electron microscope (Hitachi, Changlunake, Japan).

### 2.9. Western Blot Analysis for TLR4, NF-*κ*B, Beclin-1, and P62 Levels

The eutopic and ectopic endometrium proteins were extracted from each group. The proteins were loaded on a sodium dodecyl sulfate-polyacrylamide gel electrophoresis (SDS-PAGE), blotted to polyvinylidene fluoride (PVDF) membrane, and blocked with 5% skimmed milk in TBS for 1 h at room temperature. After TTBS washing, the primary antibody was incubated at 4°C overnight along with the washed membrane. The membranes were then incubated at room temperature for 1 hour with the secondary antibody (polyclonal goat-antirabbit HRP, 1 : 5000) after washing again with TTBS the following day. After incubation with the antibodies, the membranes were rinsed in TTBS and TBS successively, and the bands were visualized by chemiluminescence, and Quantity One gel imaging system was used to collect the pictures. GAPDH expression was used as an internal reference protein, and the relative protein expression level was analyzed by Image J software.

### 2.10. Expression Analysis of TLR4, NF-*κ*B, Beclin-1, and P62 by qRT-PCR

The total RNA from the tissues was extracted using an E.Z.N.A.® Total RNA kit II (cat. no. R6934-01; Omega Bio-Tek, Inc.) as per the manufacturer's instructions. Qualitative and quantitative analysis was carried out by UV spectrophotometer. The total RNA was reversely transcribed into cDNA using a Mon Script™ RT III all-in-one mix (cat. no. MR05101; Monad Biotech Co., Ltd.). The 44 cycles of conventional PCR were done at 95°C for 10 min, 95°C for 15 s, and 60°C for 60 s. Real-time PCR was performed in triplicate with Applied Biosystems™ 7500 Real-Time PCR system with GAPDH as a housekeeping transcript for normalization. 2^–ΔΔCT^ method was used for expression analysis as a fold chain. Shanghai Sangon Biotechnology (Shanghai, China) Co., Ltd designed and synthesized the primers. The synthetic oligonucleotide primer sequences for TLR4, NF-*κ*B, Beclin-1, P62, and GAPDH were as follows: TLR4 5′-3′ TCCACAAGAGCCGGAAAGTT and 5′-3′ TGAAGATGATGCCAGAGCGG, NF-*κ*B 5′-3′ TGTATTTCACGGGACCTGGC and 5′-3′ CAGGCTAGGGTCAGCGTATG,Beclin-1 5′-3′ AACTCTGGAGGTCTCGCTCT and 5′-3′ CGCCTTAGACCCCTCCATTC, P62 5′-3′ ATGCCTTTGGCTTTTTCGCA and 5′-3′ GGGAAAGTCCGGCAAGTGTA, and GAPDH 5′-3′ AGGAAATGATGACCTCCTGAACT and 5′-3′ TGTTTTTGTAAGTATCTTGGTGCCT.

### 2.11. Statistical Analysis

SPSS 26.0 statistical software was used for the statistical analysis; as a result of the measurement, the data are expressed as $\bar{x}$ ± *s*. For comparisons within the multiple groups, one-way ANOVA was used, while SNK tests were used for comparisons between the groups. A statistically significant difference was determined by *p* < 0.05.

## 3. Results

### 3.1. Comparison of Volume and Weight of Ectopic Foci

It was found that compared with the model group, the volume and weight of the ectopic foci in the high-dose and low-dose groups and the gestrinone group decreased, and the differences were statistically significant (*p* < 0.05 and *p* < 0.01). There were no significant differences noted between the various treatment groups (*p* > 0.05) (Table 1).

### 3.2. Comparison of Serum CA125, E_2_, P, FSH, and LH

Serum concentrations of CA125, E_2_, P, FSH, and LH were all shown to be higher in the model group compared to the sham group, with the increases being statistically significant (*P* < 0.01). BWHD high-dose, low-dose, and gestrinone groups all showed significant reductions in serum CA125, E_2_, P, FSH, and LH as compared to the model group (*P* < 0.01) (Table 2).

### 3.3. Ultrastructural Changes in the Eutopic and Ectopic Endometrium

**Figure 1:**
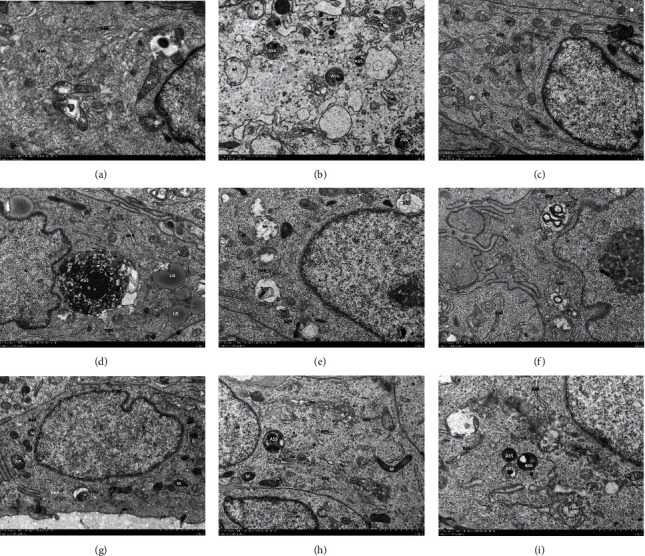
Transmission electron microscope of the endometrium. (a–e) The eutopic endometrium; (f–i) The ectopic endometrium. (a) Sham group, (b) and (f) model group, (c) and (g) BWHD high-dose group, (d) and (h) BWHD low-dose group, and (e) and (i) gestrinone group, N: nucleus; M: mitochondria; RER: rough endoplasmic reticulum; Go: Golgi apparatus; Ly: lysosome; ASS: autophagic lysosome; AP: autophagic body.

Transmission electron microscopy (TEM) revealed a low number of autophagosomes in the sham group and a significant rise in the model group. The number of autophagosomes decreased significantly after BWHD treatment, and a normal bilayer membrane structure and undegraded cytoplasm were discovered (Figure 1).

### 3.4. Expression of TLR4, NF-*κ*B, Beclin-1, and P62 Proteins by Immunofluorescence

**Figure 2:**
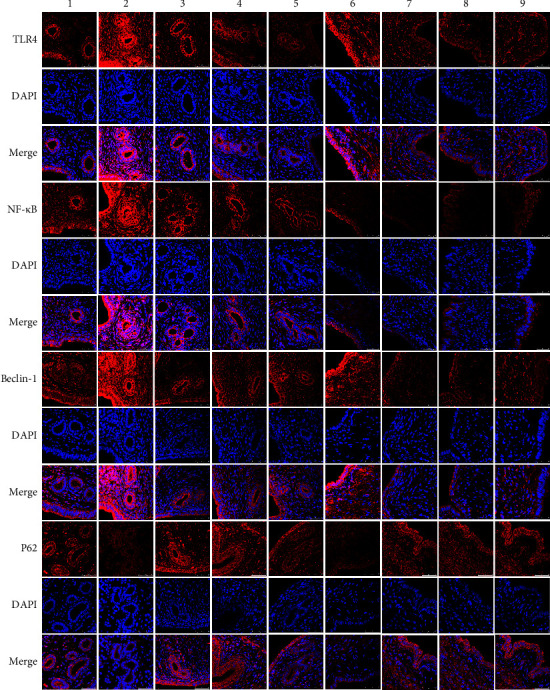
The immunofluorescence of TLR4, NF-*κ*B, Beclin-1, and P62. Note. 1–5 stands for the eutopic endometrium and 6–9 stands for the ectopic endometrium. 1 sham group, 2 and 6 model group, 3 and 7 BWHD high-dose group, 4 and 8 BWHD low-dose group, 5 and 9 gestrinone group.

The results of the immunofluorescence assay demonstrated that TLR4, Beclin-1, and P62 were primarily found in the cytoplasm, whereas NF-*κ*B was mainly localized in the cytoplasm and nucleus. The results showed that the red fluorescence of TLR4, Beclin-1, and NF-*κ*B was brighter in the model group as compared to the sham group and darker after treatment. However, the expression of P62 displayed the opposite change (Figure 2).

### 3.5. Expression of TLR4, NF-*κ*B, Beclin-1, and P62 Proteins by Western Blot

**Figure 3:**
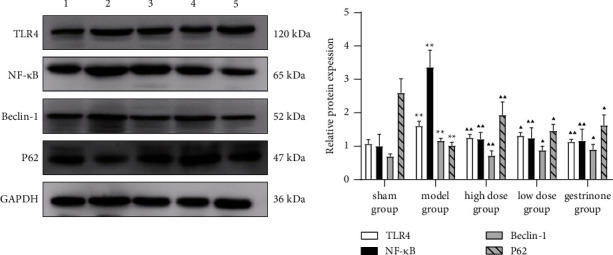
Comparison of TLR4, NF-*κ*B, Beclin-1, and P62 in the eutopic endometrium. *Note*: compared with the sham group, ^*∗*^*P* < 0.05 and ^*∗∗*^*P* < 0.01. Compared with the model group, ^▲^*P* < 0.05 and ^▲▲^*P* < 0.01.

**Figure 4:**
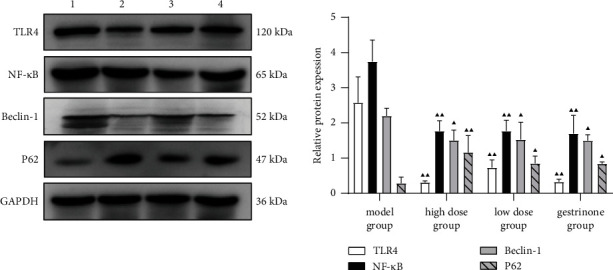
Comparison of TLR4, NF-*κ*B, Beclin-1, and P62 in the ectopic endometrium. *Note*: compared with the model group, ^▲^*P* < 0.05 and ^▲▲^*P* < 0.01.

It was observed that compared with the sham group, the expressions of TLR4, NF-*κ*B, and Beclin-1 in the eutopic endometrium of the model group were increased, while the expression of P62 was decreased (*P* < 0.05 and *P* < 0.01) (Figure 2). In addition, compared with the model group, the expressions of TLR4, NF-*κ*B, and Beclin-1 in the endometrium of the high-dose group, low-dose group, and gestrinone group were decreased, while the expression of P62 was increased (*P* < 0.05, *P* < 0.01). Moreover, compared with the model group, the expressions of TLR4, NF-*κ*B, and Beclin-1 in the ectopic endometrium of high-dose group, low-dose group, and gestrinone groups were decreased, while P62 was increased (*P* < 0.05 and *P* < 0.01) (Figures 3–4).

### 3.6. TLR4, NF-*κ*B, Beclin-1, and P62 mRNA by qRT-PCR

It was noted that compared with the sham group, mRNA levels of TLR4, NF-*κ*B, and Beclin-1 were increased in the model group, while mRNA levels of P62 were decreased (*P* < 0.05 and *P* < 0.01). In addition, compared with the model group, mRNA levels of TLR4, NF-*κ*B, and Beclin-1 in the endometrium of high-dose group, low-dose group, and gestrinone groups were decreased, and the mRNA level of P62 was increased (*P* < 0.05 and *P* < 0.01). However, compared with the model group, mRNA levels of TLR4, NF-*κ*B, and Beclin-1 in the ectopic endometrium in the high-dose group, low-dose group, and gestrinone group were decreased, while the mRNA level of P62 was increased (*P* < 0.05 and *P* < 0.01) (Tables 3 and 4).

### 3.7. Correlation Analysis of TLR4, NF-*κ*B, Beclin-1, and P62

**Figure 5:**
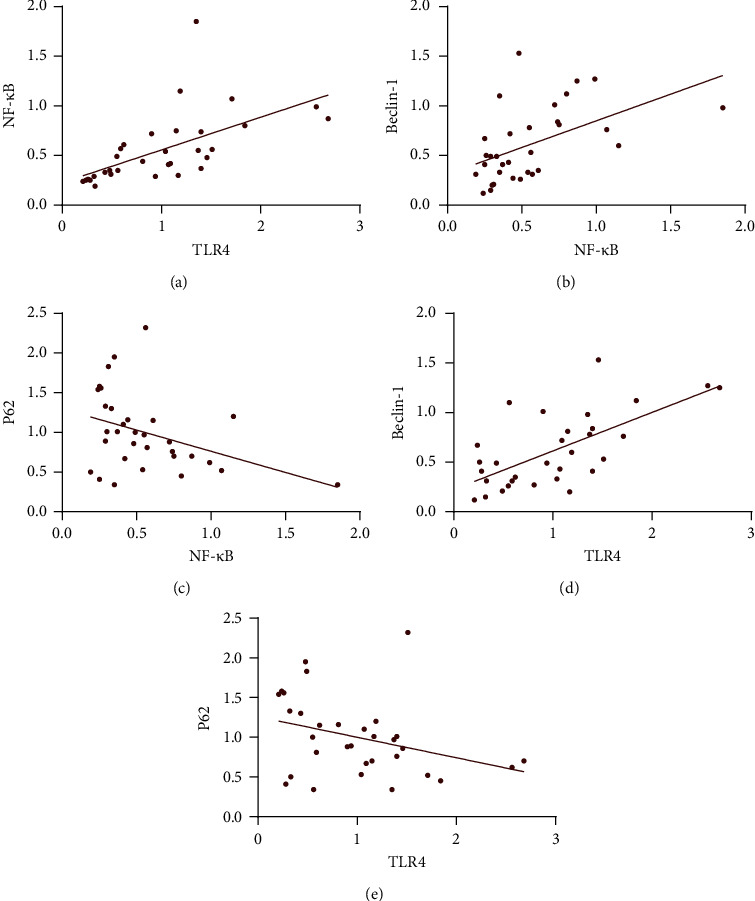
(a) Correlation analysis of TLR4 and NF-*κ*B. (b) Correlation analysis of NF-*κ*B and Beclin-1. (c) Correlation analysis of NF-*κ*B and P62. (d) Correlation analysis of TLR4 and Beclin-1. (e) Correlation analysis of TLR4 and P62.

Correlation analysis showed that TLR4 was positively correlated with NF-*κ*B, and the correlation coefficient (*R*) was 0.600 (*P* < 0.01). NF-*κ*B was positively correlated with Beclin-1, and the correlation coefficient (*R*) was 0.506 (*P* < 0.01); whereas NF-*κ*B was negatively correlated with P62, the correlation coefficient (*R*) was −0.381 (*P* < 0.05). TLR4 was positively correlated with Beclin-1, and the correlation coefficient (*R*) was 0.669 (*P* < 0.01), but there was no correlation observed between TLR4 and P62 (*P* > 0.05) (Table 5 and Figure 5).

## 4. Discussion

**Figure 6:**
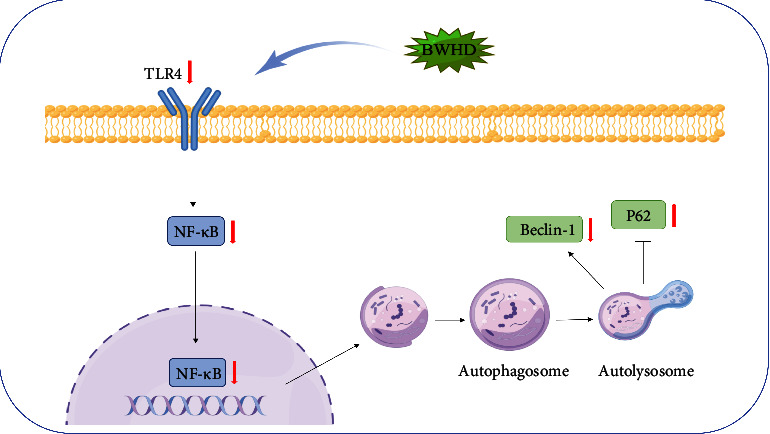
Molecular mechanism diagram of the inhibitory effect of BWHD on EMs (by Figdraw). *Note*: the protein and mRNA expressions of autophagy-related genes Beclin-1 were activated and p62 was downregulated in EMs models; BWHD may play the function of alleviating autophagy by inhibiting the TRL4/NF-*κ*B signaling pathway, thereby reducing the autophagy level, to achieve the purpose of ameliorating the progress of EMs. These results proved that there was an inhibition effect of BWHD on autophagy in EMs by regulating TLR4/NF-*κ*B signaling pathway.

In this study, we found that TLR4/NF-*κ*B signaling pathway can participate in promoting EMs pathology by increasing autophagy. Therapeutic effects against EMs can be achieved using BWHD due to its ability to effectively suppress TLR4 and NF-*κ*B expression, alleviate autophagy, decrease energy supply, and subsequently increase the cell death of ectopic lesions (Figure 6).

EMs is considered a benign gynecological illness. However, it has been shown to have growth, invasion, metastasis, and recurrence patterns comparable to malignant tumors [29]. Because of the unclear pathophysiology, there are numerous unknown aspects of EMs treatment. Western medicine mostly uses medications and surgery to treat EMs, but major side effects and recurrence rates are problems that cannot be overlooked in the treatment process. In TCM theory, EMs are classified as “zhengjia,” “dysmenorrhea,” and “infertility.” Its underlying pathophysiology is blood stasis, which is caused mostly by kidney yang insufficiency. BWHD can notify the kidney, warm Yang, remove blood stasis, and relieve pain.

In our previous research, combining clinical observation and animal experiments, we discovered that BWHD could alleviate clinical symptoms, lower VAS scores, attenuate the release of pain substances, relieve pain, reduce the size of lesions, and block the growth of EMs [27, 30]. A meta-analysis found that tonifying kidneys and activating blood stasis therapy are effective and safe for EMs, suggesting that it may be a potential method for treating EMs [31]. This study found that during a 3-week treatment period, BWHD can dramatically reduce the volume and weight of ectopic lesions while also promoting their dissipation and absorption, establishing BWHD as an effective medicine in the clinical treatment of EMs. It has a clear therapeutic effect and can effectively lower serum CA125 levels. It can also lower E_2_, P, FSH, and LH levels while also regulating hormone levels.

Ectopic lesions eliminate their foreign bodies by producing inflammatory responses, thus EMs can be thought of as a chronic immunological inflammatory illness. TLR4, belonging to the TLR family, is widely expressed on the cell membranes of immune cells as a receptor and an inflammatory mediator that can play a critical role in the signal transduction throughout the innate immune system. It is found in endometrial cells and acts as a crucial factor in the development of EMs [32]. TLR4, as we know, is essential for initiating the innate immune response through activating the different intracellular signaling pathways NF-*κ*B [33].

TLR4 signaling causes inflammation and can greatly enhance autophagosome production [34, 35]. NF-*κ*B is a crucial transcription factor that acts downstream of the TLR4 signaling pathway [36], which regulates immunological responses and is thought to be the beginning factor of several inflammatory reactions. Its activation can cause inflammation as well as the aggregation of the microtubule-associated protein light chain 3 (LC3) and the ubiquitination of Beclin-1, which can then activate autophagy [37]. The TLR4/NF-*κ*B pathway is a signal transduction system that has been linked to inflammatory immunological mechanisms and the regulation of inflammation as well as the regulation of autophagy [38]. Some researchers have discovered that the TLR4/NF-*κ*B signaling pathway can promote autophagy in the pathological phase of idiopathic pulmonary fibrosis, demonstrating that TLR4/NF-κB is required for autophagy [23].

Beclin-1 is a critical regulator of autophagy, which mediates the initiation of autophagy and is considered as a hallmark of autophagy initiation. P62 is an autophagic substrate conjunctin, which contains multiple domains and can bind to the protein to be degraded to transport it to autophagosomes, and eventually to be degraded in lysosomes [39]. P62 is an autophagy adaptor protein that can be destroyed but accumulates when autophagy is blocked. As a result, P62 is inversely associated with autophagy [40].

In this study, we assessed the expression of TLR4, NF-*κ*B, and autophagy-associated markers like Beclin-1 and P62 to see if they play a role in EMs. TLR4 and NF-*κ*B protein and mRNA levels were higher in the eutopic and ectopic endometrium of EMs animals. Furthermore, these two parameters were shown to be positively connected, suggesting that the overactivation of the TLR4/NF-*κ*B signaling pathway was associated with the incidence of EMs, and the two parameters can have a synergistic effect, which was consistent with the previous research [21]. TLR4 activation can eventually result in NF-*κ*B activation at the mRNA and protein levels, and activation of the NF-*κ*B signaling pathway can stimulate the production of numerous proinflammatory cytokines and contribute to the formation of EMs. In order to evaluate the amount of cellular autophagy, we used Western blot, qRT-PCR, and immunofluorescence to determine the expression of autophagy markers such as Beclin-1 and P62. Results showed that Beclin-1 was upregulated and P62 was downregulated in the model group and that TEM imaging revealed ultrastructural changes in both the eutopic and ectopic endometrium, including the disappearance or vacuolization of some mitochondrial cristae and an increase in the number of autophagosomes in both types of the endometrium.

A correlation study revealed that TLR4 was highly correlated with NF-*κ*B. At the same time, NF-*κ*B was also positively correlated with autophagy, suggesting that the TLR4/NF-κB signaling pathway is critical in the regulation of EMs autophagy. TLR4 and NF-*κ*B protein and mRNA expression levels reduced dramatically following BWHD treatment, demonstrating that this prescription can reduce inflammation by suppressing the activation of the TLR4/NF-*κ*B signaling pathway and thus can significantly enhance the local microenvironment. Furthermore, it was discovered that after TCM treatment, the expression of Beclin-1 was decreased considerably while that of P62 was significantly increased, implying that this prescription can reduce the level of autophagy and effectively decrease the energy supply of ectopic cells, resulting in death. It has been reported that autophagy suppression can increase apoptosis in recent years [41]. P62 activation has also been shown to suppress NF-*κ*B activation, reducing inflammation development [42]. Ravanan et al. [43] revealed that inhibiting autophagy can significantly diminish the inflammatory response generated by the NF-*κ*B pathway. Our findings suggested that BWHD could reduce autophagy and increase the shrinkage of ectopic lesions by blocking the TLR4/NF-*κ*B signaling pathway, which could be one of the mechanisms of BWHD in treating EMs. Furthermore, according to prior research [42], activation of P62 could block NF-κB activity, hence controlling the development of EMs inflammation and significantly improving the internal environment of EMs, which requires additional validation in the future.

As a result of its ability to modulate various targets and pathways with relatively fewer adverse effects, TCM is currently widely employed for the treatment and prevention of endometriosis [44]. Overall, our results show that BWHD can dramatically lower the autophagy level of abnormally increased in EMs and that the mechanism may be linked to the suppression of the TLR4/NF-*κ*B signaling pathway. By its good clinical efficacy and fewer side effects in treating EMs, Chinese herbal therapy has gained a lot of attention as a supplemental medicine. However, more research is required to determine whether TCM delays or reduces the frequency of EMs recurrence.

## Figures and Tables

**Table 1:** Comparison of volume and weight of ectopic foci in EMs rats of each group ($\bar{x}\pm$ s).

Groups	*N*	Volume (mm^3^)	Weight (mg)
Sham group	20	—	—
Model group	20	468.63 ± 67.58	345.94 ± 72.34
High-dose group	20	287.13 ± 71.20^*∗∗*^	109.76 ± 34.57^*∗∗*^
Low-dose group	20	314.67 ± 58.93^*∗*^	121.48 ± 40.57^*∗∗*^
Gestrinone group	20	246.10 ± 67.31^*∗∗*^	102.10 ± 31.90^*∗∗*^

*Note.* Compared with the model group, ^*∗*^*P* < 0.05 and ^*∗∗*^*P* < 0.01.

**Table 2:** Serum levels of CA125, E_2_, P, FSH, and LH in each group.

Groups	*N*	CA125 (U/ml)	E_2_ (pg/ml)	P (ng/ml)	FSH (mIU/ml)	LH (mIU/ml)
Sham group	20	15.22 ± 2.95	64.99 ± 12.14	48.72 ± 23.09	4.19 ± 1.17	2.76 ± 0.79
Model group	20	25.31 ± 2.93^*∗*^	82.40 ± 14.06^*∗*^	68.51 ± 28.51^*∗*^	6.01 ± 1.30^*∗*^	4.82 ± 0.97^*∗*^
High-dose group	20	17.16 ± 3.28^▲^	63.21 ± 11.12^▲^	46.99 ± 19.41^▲^	4.26 ± 0.87^▲^	2.75 ± 0.94^▲^
Low-dose group	20	19.05 ± 3.09^▲^	69.90 ± 20.87^▲^	41.59 ± 18.55^▲^	4.51 ± 1.04^▲^	2.29 ± 0.92^▲^
Gestrinone group	20	17.67 ± 3.10^▲^	61.81 ± 14.64^▲^	41.76 ± 19.88^▲^	4.28 ± 1.15^▲^	2.75 ± 0.96^▲^

*Note.* Compared with the sham group, ^*∗*^*P* < 0.01. Compared with the model group, ^▲^*P* < 0.01.

**Table 3:** Comparison of TLR4, NF-*κ*B, Beclin-1, and P62 mRNA in the eutopic endometrium.

Groups	TLR4	NF-*κ*B	Beclin-1	P62
Sham group	0.86 ± 0.30	0.85 ± 0.25	1.22 ± 0.25	1.18 ± 0.47
Model group	1.62 ± 0.65^*∗∗*^	1.66 ± 0.30^*∗∗*^	1.77 ± 0.34^*∗∗*^	0.68 ± 0.25^*∗∗*^
High-dose group	0.83 ± 0.32^▲▲^	0.68 ± 0.26^▲▲^	0.69 ± 0.20^▲▲^	1.12 ± 0.28^▲^
Low-dose group	0.94 ± 0.42^▲▲^	1.21 ± 0.38^▲▲^	0.97 ± 0.35^▲▲^	0.93 ± 0.21
Gestrinone group	0.58 ± 0.24^▲▲^	0.61 ± 0.23^▲▲^	0.61 ± 0.29^▲▲^	1.08 ± 0.43^▲^

*Note.* Compared with the sham group, ^*∗∗*^*P* < 0.01. Compared with the model group, ^▲^*P* < 0.05 and ^▲▲^*P* < 0.01.

**Table 4:** Comparison of TLR4, NF-*κ*B, Beclin-1, and P62 mRNA in the ectopic endometrium.

Groups	TLR4	NF-*κ*B	Beclin-1	P62
Model group	1.67 ± 0.70	0.94 ± 0.46	1.08 ± 0.30	0.63 ± 0.29
High-dose group	0.92 ± 0.38^▲▲^	0.42 ± 0.13^▲▲^	0.40 ± 0.15^▲▲^	1.18 ± 0.55^▲^
Low-dose group	1.04 ± 0.37^▲▲^	0.56 ± 0.17^▲▲^	0.67 ± 0.23^▲▲^	0.93 ± 0.22^▲^
Gestrinone group	0.37 ± 0.12^▲▲^	0.30 ± 0.09^▲▲^	0.29 ± 0.13^▲▲^	1.27 ± 0.58^▲▲^

*Note.* Compared with the model group, ^▲^*P* < 0.05 and ^▲▲^*P* < 0.01.

**Table 5:** Correlation analysis of TLR4, NF-*κ*B, Beclin-1, and P62.

Values	TLR4 vs. NF-*κ*B	NF-*κ*B vs. Beclin-1	NF-*κ*B vs. P62	TLR4 vs. Beclin-1	TLR4 vs. P62
*r* value	0.600	0.506	−0.381	0.669	−0.336
*P* value	0.001	0.003	0.031	0.001	0.060

## Data Availability

The datasets used and analyzed during the current study are available from the corresponding author upon reasonable request.
